# The Role of E6 Spliced Isoforms (E6*) in Human Papillomavirus-Induced Carcinogenesis

**DOI:** 10.3390/v10010045

**Published:** 2018-01-18

**Authors:** Leslie Olmedo-Nieva, J. Omar Muñoz-Bello, Adriana Contreras-Paredes, Marcela Lizano

**Affiliations:** 1 Unidad de Investigación Biomédica en Cáncer, Instituto Nacional de Cancerología, México/Instituto de Investigaciones Biomédicas, Universidad Nacional Autónoma de México, Av. San Fernando No. 22, Col. Sección XVI, Tlalpan, 14080 Mexico City, Mexico; leslie_azul25@hotmail.com (L.O.-N.); omarmube@gmail.com (J.O.M.-B.); adrycont@yahoo.com.mx (A.C.-P.); 2 Departamento de Medicina Genómica y Toxicología Ambiental, Instituto de Investigaciones Biomédicas, Universidad Nacional Autónoma de México, 04510 Mexico City, Mexico

**Keywords:** HPV, E6, splicing, E6*, spliceosome

## Abstract

Persistent infections with High Risk Human Papillomaviruses (HR-HPVs) are the main cause of cervical cancer development. The E6 and E7 oncoproteins of HR-HPVs are derived from a polycistronic pre-mRNA transcribed from an HPV early promoter. Through alternative splicing, this pre-mRNA produces a variety of E6 spliced transcripts termed E6*. In pre-malignant lesions and HPV-related cancers, different E6/E6* transcriptional patterns have been found, although they have not been clearly associated to cancer development. Moreover, there is a controversy about the participation of E6* proteins in cancer progression. This review addresses the regulation of E6 splicing and the different functions that have been found for E6* proteins, as well as their possible role in HPV-induced carcinogenesis.

## 1. Introduction

Cervical cancer continues to be a major public health problem, being the fourth cause of cancer mortality among women worldwide [1]. The persistent infection with High-Risk Human Papillomavirus (HR-HPV) is the main risk factor associated with cervical cancer development [2]. HPV sequences have been detected in almost 99% of the analyzed cervical cancer biopsies [3,4]. Moreover, HPV has also been linked to other anogenital [5,6] and oropharyngeal cancers [7].

Hitherto, more than 200 HPV types have been identified [8,9], which differ in more than 10% of nucleotide sequences within the L1 gene [10]. Commonly, HPVs infect basal layer cells of epithelia and are classified as cutaneous or mucosal types, being the infections with mucosal HPVs the most frequent sexually transmitted diseases worldwide [11]. From approximately 40 HPV types that infect the anogenital mucosal epithelium, 15 types are the most commonly found in cancer biopsies and thus, have been classified as HR-HPVs: HPV16, 18, 31, 33, 35, 39, 45, 51, 52, 56, 58, 59, 68, 73 and 82. Low-Risk HPV (LR-HPV) types are mainly related to mild dysplasia or genital warts [12]. HR-HPV16 and 18 are the most prevalent HR types, found in close to 60% and 15% of cervical cancer cases, respectively. LR-HPV6 and 11 are the most frequent types found in warts [13].

Human Papillomaviruses are small non-enveloped viruses of 55 nm, containing a circular double-stranded DNA of approximately 8 kb in length. The HPV genome is divided into three regions: the long control region (LCR) that regulates transcription and replication, the early region harboring nucleotide sequences of six common genes (E6, E7, E1, E2, E4 and E5) expressed in a primary infection [14] and the late region that contains open reading frames (ORFs) encoding the L1 and L2 structural proteins involved in viral encapsidation [15].

The main viral oncoproteins E6 and E7 regulate cell cycle progression by promoting the degradation of the tumor suppressor proteins p53 and pRb, respectively. These and other interactions affect cellular pathways leading to malignant transformation [16,17].

Multiple HPV genes are expressed in a polycistronic pre-mRNA from a single strand. Depending on the state of differentiation of the epithelial layers, early or late promoters are activated leading the transcription of the early and late regions as polycistronic mRNAs. These transcripts are polyadenylated at sequences termed early and late polyadenylation sites, which are located downstream of each polycistronic mRNA [18,19].

Several transcripts are produced throughout an HPV infection by alternative splicing, which generates different mRNA expression patterns [20]. Alternative splicing within E6-E7 ORFs is a common feature of HR-HPVs, while no splicing in this region has been detected among LR-HPVs [21]. Full-length E6 from HR-HPV types is expressed from mRNAs with no splicing within E6 ORF, while E7 can be transcribed from different mRNAs including those with splicing in E6 [22,23]. The splicing process produces several transcripts containing E6 truncated mRNAs named E6*, which are derived from a donor splicing site within the E6 ORF and one of the different acceptor sites located in the early mRNA [24]. The most abundant E6 truncated mRNA is termed E6*I, which is a poorly studied protein. E6*I shares approximately the first 44 aa with the E6 full-length protein (E6) and the intron removal promotes a change in the E6 ORF adding approximately 13 aa that are only contained in E6*I isoform and generating a new stop codon [25].

This review focusses on the transcription patterns of E6/E6* and their regulation in different models, as well as the controversial roles of E6* proteins that affect cellular processes. The evidences and controversies represent an opportunity for the study of E6* proteins in order to establish their participation in the HPV life cycle and/or in the initiation or progression of cancer.

## 2. HPV Life Cycle

The HPV life cycle depends on differentiation and replication of the host-infected cells and is characterized by having two phases: latent infection, where the episome is replicated and maintained and productive infection, where the late proteins are produced and virions are formed [26].

Depending on HPV type, multiple entry pathways have been suggested. Generally, HPVs infect the undifferentiated basal cells of the epithelium through a micro-wound. Additionally, the accessibility of cells that are close to the squamo-columnar junction increases the possibility of HPV infection of this single cell layer [27]. The precise mechanism and receptors used by HPV to infect the epithelial cells are poorly known. The most accepted models for HPV16 suggest that the HPV L1 capsid protein attaches to heparan sulfate proteoglycans (HSPGs) [28] inducing conformational changes in the capsid and transferring the viral particle to a secondary non-HSPG entry receptor [29]. This transfer is facilitated through cleavage of the L2 protein by the convertase furin [30]. In contrast to the use of pseudovirus models, some analyses with native viruses have shown that the heparan sulfate receptor and furin cleavage activity are not required for all HPV types [31,32,33].

Several secondary L1-specific receptors have been proposed to mediate the infection, such as α-6 integrin [34], keratinocyte growth factor receptor (KGFR), epidermal growth factor receptor (EGFR) [35] and tetraspanins [36]. Finally, an L2-specific receptor, the S100A10 subunit of the annexin A2 heterotetramer, is thought to be involved in promoting viral entry [37].

After viral attachment to the host cell, the endocytic uptake of HPV implies a non-canonical internalization pathway related to micropinocytosis dependent on actin dynamics [38]; however, the precise cellular components mediating HPV uptake into host cells remain unknown.

Following virus entry, the viral capsid binds to Sortin nexin 17 at the endosomal compartments, which seems to help the L2-DNA complex to escape from the lysosome [39] and finally travel to the nucleus via dynein-mediated transport along microtubules [40].

In the latent phase, low levels of E1, E2, E6 and E7 are expressed in undifferentiated basal cells, where normal differentiation is retarded. During this phase, low replication rate occurs generating approximately 50–100 viral genomes per cell [41]. Further, in the proliferative phase, E6 and E7 are highly expressed from the middle to the upper layers of the differentiating epithelium [42]. The E2 protein recruits E1, a viral DNA helicase, to its binding site in the viral origin of replication, facilitating viral DNA replication and leading to the production of thousands of viral genome copies per cell in differentiated keratinocytes [26,43]. E4 stabilizes E2 and facilitates nuclear localization of E1, increasing E1/E2 dependent viral genome amplification [44]. Moreover, E2 acts as transcriptional factor controlling the expression of viral genes, through the recruitment of cellular factors to the LCR, promoting the activation or repression of viral transcription [43].

Finally, the viral life cycle is completed by the synthesis of L1 and L2 proteins in the uppermost layer of the epithelium, allowing the encapsidation of newly replicated genomes and the release of mature virions [27].

Most of the HPV infections are transient and cleared by the immune system in less than two years. Furthermore, when clinical lesions are generated, the majority undergo spontaneous regression [45]. It has been proposed that a determinant key to neoplastic progression is the persistent infection by HR-HPVs, which after a long time could lead to genomic instability and to viral genome integration into the host genome, at this stage, no viral progeny is produced [46]. As an episome, viral early gene expression is controlled by E2 but when integration occurs, E2 gene expression is commonly disrupted, leading to an increase in the expression of E6 and E7. The formation and maintenance of tumors needs the constant expression of E6 and E7 oncoproteins [47].

In cervical cancer biopsies, the HR-HPV genome is commonly found integrated, although in a small proportion of the cases HPV-DNA remains as an episome but at a high copy number [48]. It has been proposed that in HPV episomes, E2 binding sites contained in the LCR can be methylated preventing the E2 transcriptional repression and allowing the overexpression of E6 and E7 oncoproteins [49]. This indicates that HPV integration in some cases may not be a requirement for cellular transformation.

## 3. The Splicing Process

The splicing process is an essential mechanism that regulates gene expression and contributes to cell proteomic diversity. During transcription, RNA polymerase II generates a pre-mRNA that harbors exonic and intronic *cis* regulatory elements, able to recruit the spliceosome complex. The spliceosome regulates the exon-exon junction, generated when the intronic sequences are released, which is a crucial step in the maturation of the pre-mRNA [50,51]. The spliceosome is formed by a variety of small nuclear RNAs (U1, U2, U4/U6 and U5) organized in small nuclear ribonucleoproteins (snRPNs), complexed to several regulatory proteins [52,53]. The spliceosome complex assembly is directed by consensus sequences that flank exon-intron joints at the 5′ donor site ((C/A)AGGU(A/G)AGU) and 3′ acceptor site ((C/U)AG) of the pre-mRNA, in addition to intronic sequences termed branch points ((C/U)NC/U)U(A/G)A(C/U)) and a polypyrimidine tract [54]. Moreover, the pre-mRNA harbors auxiliary *cis*-acting elements termed exonic/intronic splicing enhancers (ESEs and ISEs, respectively) and exonic/intronic splicing silencers (ESSs and ISSs, respectively) that regulate splicing through the binding with regulatory proteins that stimulate or repress the spliceosome complex assembly [54].

**Figure 1:**
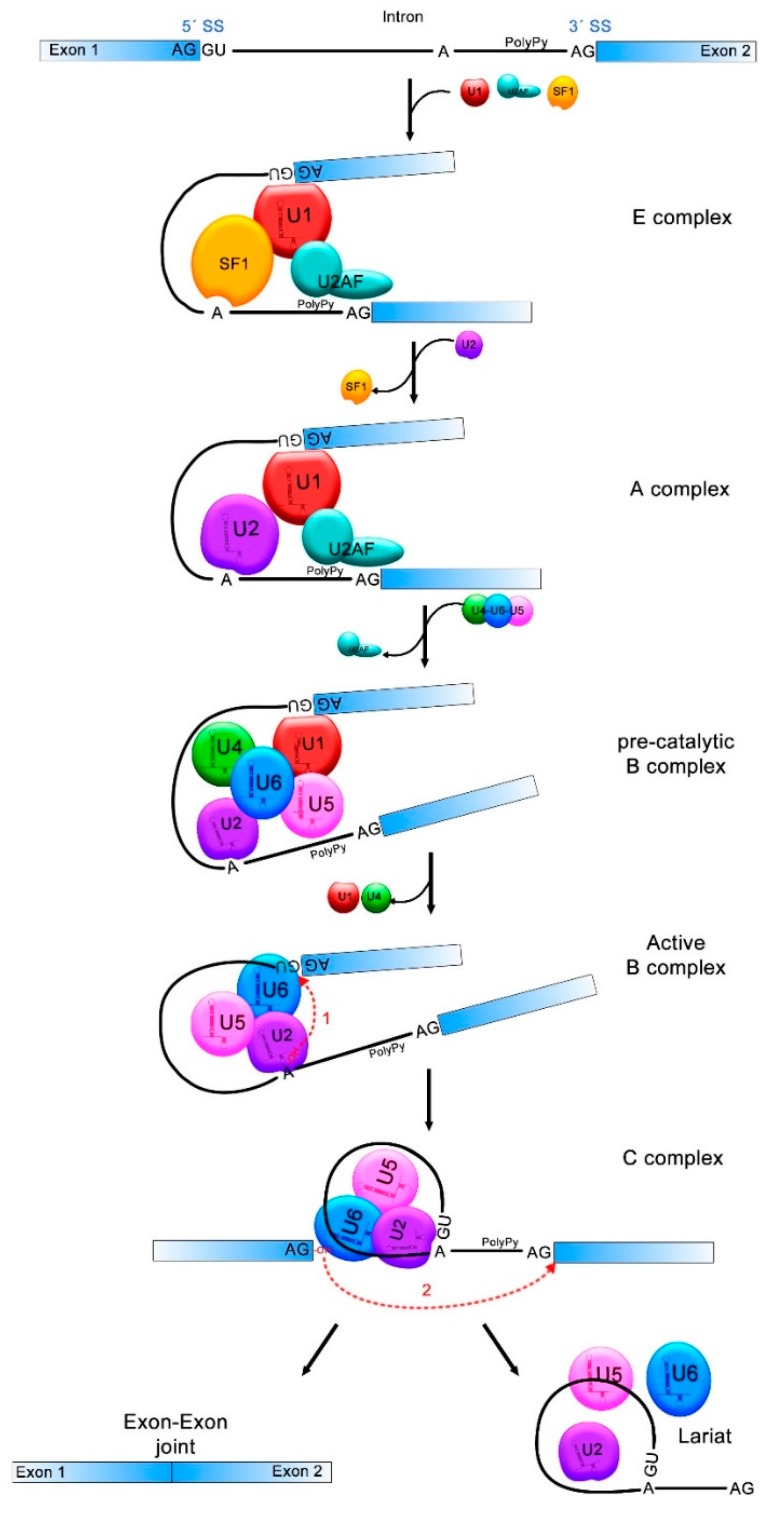
pre-mRNA splicing process. Donor and acceptor splicing sites (5′SS and 3′SS) in the exon-intron junctions, the branch point (A) and the polypyrimidine tract (PolyPy) are contained in the pre-mRNA. In the E complex, the U1 small nuclear ribonucleoprotein (snRNP) binds to the 5′SS, the splicing factor 1 (SF1) to the branch point and the U2 Auxiliary Factor (U2AF) to the PolyPy, approaching the 5′SS and 3′SS. In the A complex, U2 associates to the branch point and SF1 is disassembled. U4/U6/U5 complex binds and U2AF is released from the spliceosome in the pre-catalytic B complex. The active B complex is formed when U1 and U4 exit from the spliceosome and structure rearrangements induce the first splicing reaction where the phosphodiester bond at the 5′SS is attacked by the 2′-OH of the A forming a lariat structure. In the C complex, the second reaction forms a bond between the 3′-OH of the first exon and the 5′-P of the second exon. The intronic sequences are discarded and exons 1 and 2 come together. The transitions between one and other splicing complex are indicated with black solid arrows and the two splicing reactions are indicated with red dotted arrows.

Briefly, the U1 small nuclear ribonucleoprotein (snRNP) binds to the 5′ splice site, allowing the binding of the splicing factor 1/mammalian branch point binding protein (SF1/mBBP) to the branch point and the interaction of the U2 Auxiliary Factor (U2AF) with the polypyrimidine tract, forming the E complex which approaches the 5′ and 3′ splicing sites. Then, the U2 snRNP associates with the branch point and induces the displacement of SF1/mBBP, leading to the formation of the A complex. Later, the pre-assembled complex, U4/U6/U5 tri-snRNP, is recruited, generating the pre-catalytic B complex. In this step, all snRNPs are catalytically inactive and require other rearrangements to induce the first splicing reaction. Afterwards, U1 and U4 are removed from the B complex while U2, U5 and U6 are rearranged, forming the active B complex. This complex is then catalytically activated by the DEAH (Asp-Glu-Ala-His)-box RNA helicase Prp2 (catalytically activated B complex). In this step, the phosphodiester bond at the 5′ splice site (exon-intron joint) is attacked and broken by the 2′-OH of the adenosine at the branch point, which creates a new bond between the 5′ side of the intron and the adenosine, forming the lariat structure. At this point the C complex is formed, which induces the catalysis of the second bond between the 3′-OH of the first exon and the 5′ acceptor site of the second exon (exon-exon joint). Finally, the intronic sequences are discarded, the exons come together and the spliceosome is disassembled [20,54,55] (Figure 1).

Since the consensus sequences in splicing sites are not well conserved, the nucleotide combinations increase the possibility of multiple choices of splice sites within the pre-mRNAs, which leads to selective intron and exon removal, allowing the expression of a great variety of isoforms derived from a single pre-mRNA. This process is termed alternative splicing [20].

**Figure 2:**
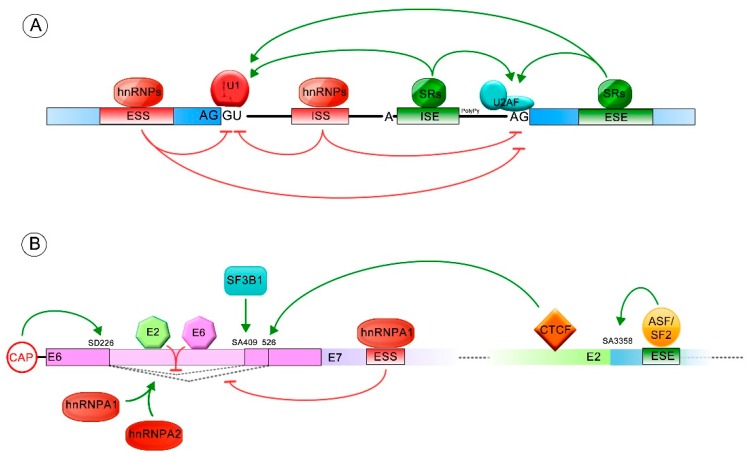
Splicing regulation. Green arrows indicate positive splicing regulation, while red arrows represent negative splicing regulation. (**A**) General regulation mediated by *cis* and/or *trans* elements is shown. The exonic and intronic splicing enhancers (ESE and ISE) frequently stimulate the splicing process by binding to serine/arginine-rich proteins (SR proteins). The exonic and intronic splicing silencers (ESS and ISS) commonly repress the splicing process, through binding with heterogeneous ribonucleoproteins (hnRNP) regulatory proteins; (**B**) Splicing regulated by *cis* and *trans* acting elements, allowing formation of different E6/E6* transcript patterns. The ESS and ESE sequences (exonic splicing silencer and enhancer, respectively) and the splicing donor (SD) and acceptor (SA) sites involved in E6 splicing regulation are also shown.

In addition, exonic and intronic splicing enhancers (ESEs and ISEs) and/or silencers (ESSs and ISSs) are required to regulate the splicing process: negatively, by the interaction with the heterogeneous ribonucleoproteins (hnRNPs) and positively, with serine/arginine-rich protein (SR). The hnRNPs (i.e., hnRNPA1 and hnRNPA2) bind mainly to the silencer elements, blocking the recognition of the exon-intron junctions by elements of the spliceosome. In contrast, the SR proteins (SRF1-12) usually bind to the enhancer sequences, acting as general activators of exon definition. The contribution of the SR and hnRNP proteins defines the overall recognition potential of an exon and/or the affinity for the spliceosome [20,52,56,57] (Figure 2A).

## 4. Splicing within HR-HPV E6

The LCR contains cellular and viral transcription factor binding sites, as well as transcriptional enhancers, a replication origin, a late polyadenylation site and late regulatory elements [19,27]. The early promoter is located upstream of the E6 ORF (p105 for HPV18 and p97 for HPV16) and is responsible for early gene transcription. The late promoter that resides inside of the E7 ORF, drives E4, L1 and L2 gene expression. Other sequences that could act as possible promoters have been described but their functions are not clearly understood.

In low-risk HPVs the E6 and E7 genes are transcribed from two independent promoters, while in high-risk HPVs those genes are transcribed as a single polycistronic pre-mRNA from the early promoters. A common feature of high-risk HPVs is that the E6/E7 polycistronic mRNA contains at least one donor and one acceptor splicing site that can trigger the alternative splicing process, inducing the expression of a variety of E6 spliced transcripts termed E6* [18,58]. In contrast, low-risk HPVs and beta-papillomavirus types do not undergo splicing in this region [21].

Depending on the HR-HPV type, different transcripts are derived from one of the donor splicing sites contained in the E6 ORF and one of the acceptor splicing sites located within E7, E2 or E4 ORFs. The splicing pattern of HPV type 16 has been thoroughly studied and the following spliced transcripts have been identified: E6*I, E6*II, E6*III, E6^E7, E6^E7*I, E6^E7*II, E6*IV, E6*V and E6*VI [18,59,60,61,62]. Conversely, the described transcripts for HPV18 are: E6*I, E6*II, E6*III, E6^E7 [60,63,64]. Less is known about transcripts resulting from splicing in the E6 pre-mRNA of other HR-HPV types, such as HPV31 having E6*I and E6^E4; HPV33 with E6*I, E6*II and E6*III; and HPV58 with E6*I and E6*II [65,66,67,68]. For other HPV types only the E6*I transcript has been detected, although the existence of other E6 spliced transcripts cannot be discarded [21,69]. Donor and acceptor sites for the identified different transcripts are depicted in Table 1.

Interestingly, it has been proposed that E6 nucleotides 226 and 409 (donor and acceptor sites, respectively) from HPV16 are preferentially selected among other splicing sites, leading to the release of intron I, generating E6*I [61]. A suboptimal branch point sequence was previously identified within intron I of HPV16 (AGUGAGU) which contains the 328G instead of the typical adenosine [72]. An optimal branch point was further discovered within the same intron (AACAAAC), proposed to be the preferred branch point sequence, where 385A allows the selection of E6*I and expression of E7 [61].

## 5. E6/E6* Transcription Patterns

**Figure 3:**
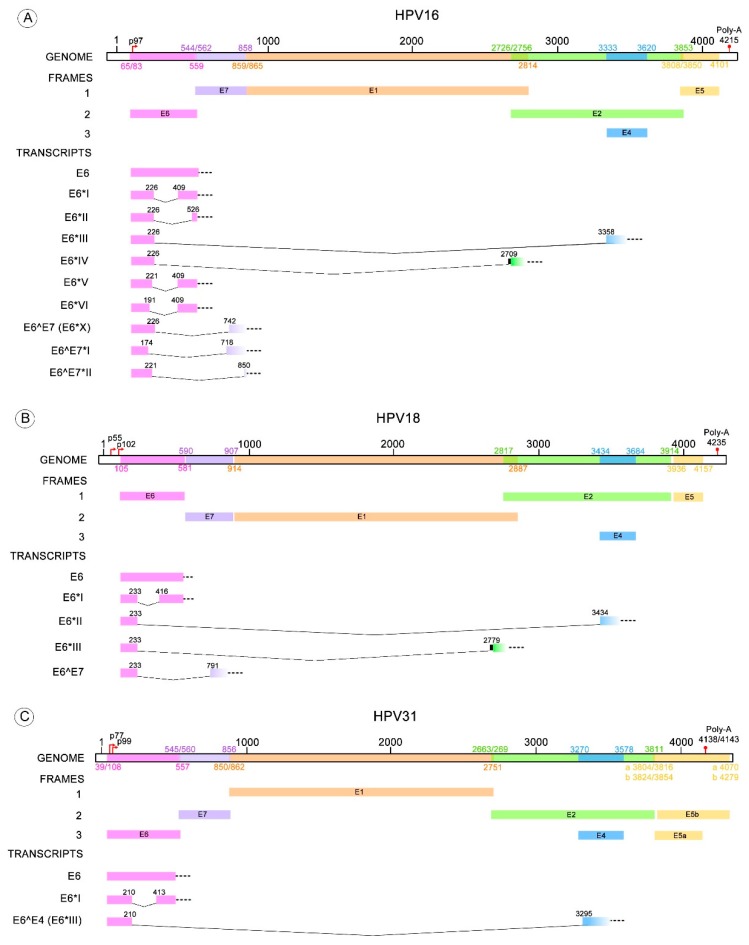

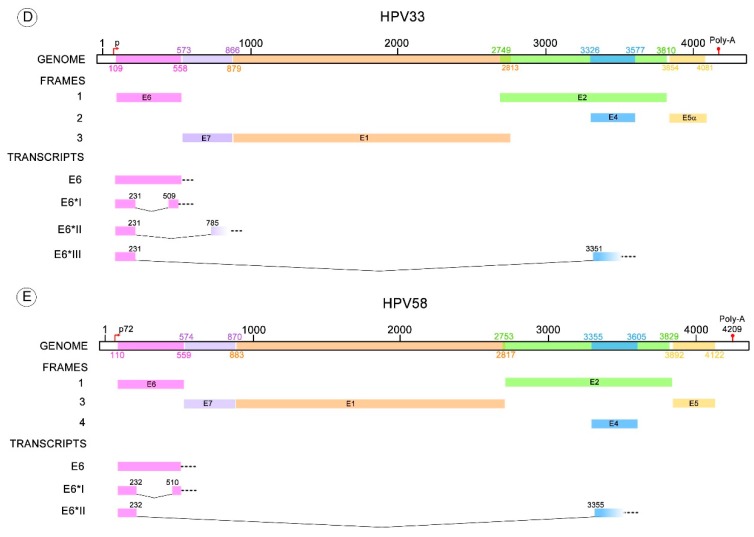
HR-HPV E6 alternative RNA splicing. The donor and acceptor splicing sites for the E6 truncated transcripts of different HPV types that possess more than one variant of E6* mRNA. (**A**) The E6* transcripts identified in HPV16 are E6*I to VI, E6^E7, E6^E7*I and E6^E7*II; (**B**) Four E6* transcripts have been described for HPV18 termed E6*I to III and E6^E7; (**C**) E6*I and E6^E4 transcripts are known for HPV31; (**D**) E6*I to III for HPV33; (**E**) E6*I and II transcripts for HPV58. All these transcript variants contain a donor splicing site within the E6 open reading frame (ORF), while the acceptor splicing site is contained through the early HPV pre-mRNA (E6, E7, E2 or E4 ORFs). The nucleotide positions of early promoters (p) and early polyadenylation (Poly-A) sequences, as well as the positions of early genes, were obtained from Papillomavirus episteme [8]. The early promoter of HPV58 was obtained from Li Y. et al. 2013 [67]. All the donor and acceptor splicing sites are listed in Table 1.

Many studies have described the E6/E6* patterns found in cervical cancer cell lines with endogenous expression of HPV or in cells with ectopic expression of HPV sequences. These patterns have also been identified in HPV infected biopsies with normal or altered cytology and in HPV-related cancers. Most of those studies are focused on the expression patterns of HPV16 and HVP18; although, information is available for other HR-HPV types such as, 31, 33 and 58 (Figure 3) [65,66,67].

The donor and acceptor splicing sites necessary to generate E6*I were described for the first time in the HPV16 positive CaSki cell line [73]; however, this isoform was first named E6* in a study performed using the HPV18 positive HeLa cell line [74].

E6/E7 splicing patterns have been recognized by different methods in a variety of HR-HPV containing cell lines and those studies consistently reveal the presence of higher amounts of E6*I mRNA compared to the E6 transcript [74,75,76,77]. In addition, the E6*II transcript is usually present in higher amounts than E6 but at lower levels than E6*I [75].

It has been demonstrated that E6*I is highly expressed in a model of HPV primary infection, where the replication cycle of HPV18 is efficient [63]; suggesting that the expression of E6*I could have an important role in the first stages of viral infection.

Moreover, studies in W12—cells derived from a low grade cervical lesion with episomal HPV16—showed that while E6 mRNA was not detected, E6*I and E6*III were expressed [59]. In further studies, different subclones were isolated from the W12 cell line, generating a W12-derived model of cervical tumor progression. Such clones contain different physical states of the HPV16 genome (episomal or integrated), exhibiting different biological outcomes: differentiated non-tumorigenic, less differentiated non-tumorigenic, tumorigenic and invasive cells. Interestingly, all of these cell lines express E6, E6*I and E6*II transcripts but the carcinogenic clones showed a significant increase in the expression of all E6 transcripts, in addition to the expression of the E6*X [70]. These findings suggest that the E6/E6* expression patterns could be independent of the physical state of the HPV genome but dependent on the lesion grade.

The E6/E6*I transcription patterns were evaluated in 12 oncogenic and 11 possibly-oncogenic HPVs, where E6/E6*I were found to be expressed in the majority of those HPV types, although with different patterns. In contrast to several studies, this report shows that E6*I transcript from HPV16 and 18 were present in lower amounts than E6 [21]. It was previously demonstrated that the distance between the 5′ Cap site and the intron is rate limiting for E6 RNA splicing [78]. Therefore, changes in the proportion of E6/E6* observed in different studies could be partially explained by the 5′ added nucleotide sequences in the E6 expressing vectors, which increase the distance between the E6 intron and the 5′ Cap in the pre-mRNA.

E6 and E6-spliced mRNAs have been investigated in patient samples, aiming to find a correlation with different stages during transformation. Many studies show that premalignant or malignant cervical and oropharyngeal lesions positive for HPV16 genomes, exhibit higher amounts of E6*I than E6, similar to the results described in cell lines [77,79,80]. Other studies detected E6*I and E6*III transcripts in cervical cancer, as well as in low and high-grade lesions, where no E6 mRNA was identified, maybe due to the different sensitivities of the technical approaches used [81]. In HPV16 positive cervical cancer biopsies, the proportion of E6, E6*I, E6*II and E6^E7 transcripts varies but E6^E7 is consistently present at lower levels, while the expression of E6*I is the highest [60]. Furthermore, the levels of HPV16 E6, E6*I and E6*II mRNAs are higher in cervical cancer samples compared to those in oropharyngeal cancer [82], suggesting that cellular contexts could be involved in the expression of HPV sequences.

Through RNA-seq quantitative sequencing, the proportion of HPV transcripts in cervical samples has been determined. In Cervical Intraepithelial Neoplasia grade 3 (CIN3) and Squamous Cervical Cancer (SCC), low levels of E6 transcripts were found, representing less than 5% of all HPV transcripts in each sample; conversely, E6*I represented close to 5%, 40% and 50% of all HPV mRNAs in Cervical Intraepithelial Neoplasia grade 2 (CIN2), CIN3 and SCC samples, respectively [24].

Controversial results about the association between the expression of E6*I/E6*II and the grade of cervical lesions have been reported. A positive association between higher concentrations of E6*I and E6*II transcripts and high-grade cervical lesions, as well as cervical cancer, was found, being E6*I the most abundant of these mRNAs [82,83]. In contrast, another study did not reveal differences in E6*I levels in the different lesion grades but a significant decrease of E6*II was observed in high-grade lesions [84]. Moreover, some studies have proposed E6*II expression as an indicator of cervical neoplasia severity [85]. These results show that the association between E6*I/E6*II patterns and lesion grade cannot be confirmed at this moment.

E6* expression has also been studied in uncommon HPV-related cancers. In squamous cell scrotal cancer samples, HPV16 E6*I transcripts were found [86]. Additionally, in breast tumor samples infected with HPV16, E6*I, E6*II, E6^E7 (E6*X), E6^E7*I and E6^E7*II transcripts were detected [62]. These results suggest that HPV is transcriptionally active in those tumor samples.

It is worth mentioning that variations in 2 to 5% of genomic sequences within the same HPV type are defined as intra-type variants, which have been associated with distinct biological outcomes of HPV infections [87]. It has been reported that nucleotide changes within HPV18 E6 variant genes (Asian-Amerindian, European and African phylogenetic branches) result in different E6/E6*I splicing patterns in MCF-7 cells and cervical tumor biopsies. Interestingly, the cells and tumors harboring the Asian-Amerindian variant of E6 expressed higher levels of E6 than E6*I, while those with the African variant exhibited a higher proportion of E6*I [88,89]. Furthermore, European variants of HPV16 do not exhibit differences in E6/E6* splicing patterns [90].

In conclusion, even when E6/E6* patterns differ in pre-malignant lesions and cancer, E6*I is the transcript present in higher amounts. Moreover, it seems that all transcript levels increase as the lesions progress to cancer. This effect could be related to an increase in HPV transcription and/or replication rates, which might allow the detection of those spliced transcripts found at low levels. However, further studies are needed to confirm this statement.

## 6. Regulation of E6/E6* Patterns

Alternative splicing of HPV transcripts increases the complexity of viral gene expression. The E6/E6* patterns change through the cell cycle, being the E6*I transcript more abundant than E6 during G2/M phase [91]. Several regulators have been identified that control transcription, splicing and polyadenylation of early and late mRNAs. However, few *cis* and *trans* acting regulators have been found to modulate E6/E6* splicing patterns (Figure 2B) [19,20,92,93].

The serine/arginine-rich splicing factor 1, 2 and 3 (SRSF1, 2 and 3) are augmented in HPV16 positive cervical cancer cell lines compared with HPV16 positive non-tumorigenic cells. These proteins increase E6/E7 mRNA stability and protect E6 transcript from decay. Interestingly, E6/E6* splicing is not affected by the SRSF overexpression [70].

Using a raft culture model, it has been shown that CCCTC-Binding Factor (CTCF) can bind to E2 ORF of HR-HPV types and induce an increase of E6*II mRNA without affecting other E6 spliced transcripts [94].

The ASF/SF2 splicing factor interacts with an HPV16 splicing enhancer located downstream of the SA3358 site, promoting splicing particularly at this acceptor site. SA3358 site allows the production of E6*III if the SD226 site is selected but can also produce other E6* mRNAs with the SD880, promoting an increase in all of the E6 spliced transcripts [95,96].

The SF3B1 splicing factor has also been reported to increase HPV16 E6 mRNA splicing, favoring the E6*I isoform [97]. Head and neck cancer cells positive for HPV16 were treated with meayamycin B, a potent inhibitor of SF3B1, showing a decrease in the levels of E6*I mRNA with an increase of the full-length E6 transcript. When SF3B1 was knocked down, similar effects were observed, demonstrating that the biogenesis of E6*I is influenced by SF3B.

The splicing at the SD226 site is favored when E6/E7 mRNAs are capped through the interaction with Cap binding factors. When the distance from 5′mRNA Cap to the SD226 is increased, the levels of E6 are higher, while a distance less than 307 nucleotides seems to be optimal to promote the splicing at SD226, facilitating E6*I expression [78].

Together, hnRNPA1 and hnRNPA2 promote splicing of E6 HPV16 mRNA. In contrast to hnRNPA1 only, that in the presence of Epidermal Growth Factor (EGF) induces an increase in un-spliced E6 mRNA [98]. This evidence could be associated to the exonic splicing silencer (ESS) within the E7 ORF, which contains an hnRNPA1 binding motif that reduces 233^416 splicing (E6*I) and induces E6 expression in HPV18-transfected or -infected cells [64].

Upon activation of EGFR and Erk1/2 MAPK by EGF, E6/E7 splicing is reduced. Although the exact mechanism has not yet been described, it is proposed that this effect could be mediated through regulators controlled by growth factor pathways, such as Brm and Sam68, which increase the levels of E6/E7 mRNA in the presence of EGF [98].

Interestingly, HPV proteins also modulate E6/E7 mRNA splicing by acting as RNA binding proteins. E2 and E6 proteins bind to intron 226–409 and might interfere with the cellular splicing machinery, decreasing the levels of E6*I transcript in HPV16 infected cells. This reduction could be carried out by SR proteins through their interaction with E2 and E6 viral proteins [99]. Therefore, expression of E6, E6* and E7 can be affected by the different splicing regulatory proteins, depending on their availability during cellular differentiation or cancer progression.

## 7. E6* Related Functions

**Figure 4:**
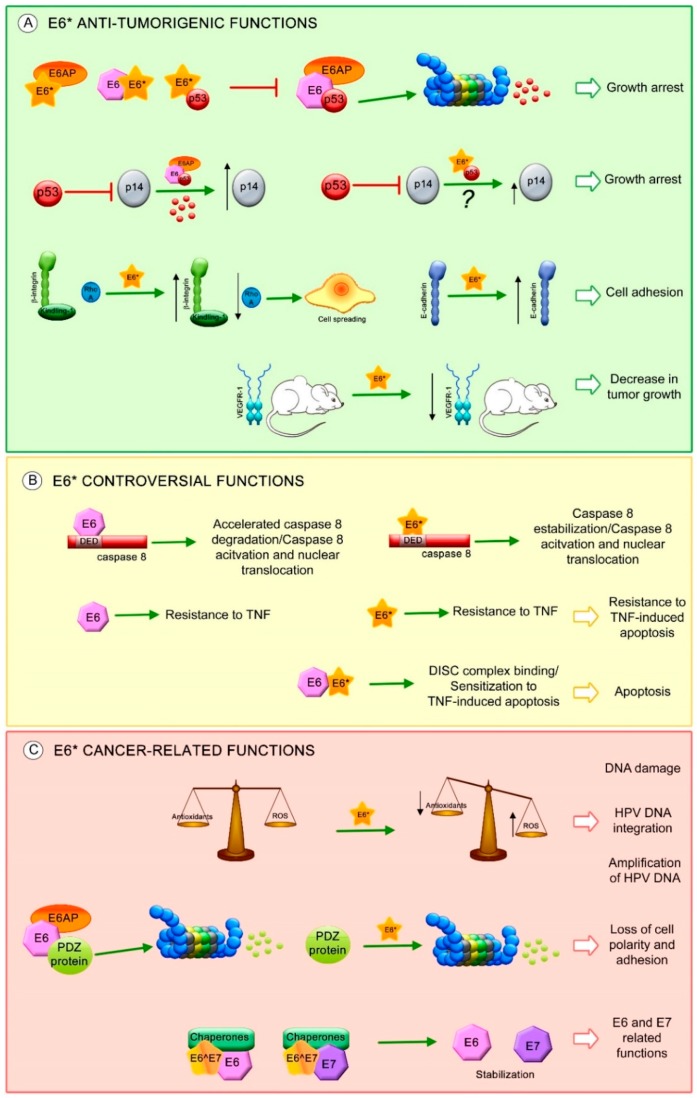
E6* isoform-related functions. Proposed E6* functions involved in (**A**) anti-tumorigenic effects, such as: increase in growth arrest through inhibition of E6-mediated p53 degradation and increase in p14 protein levels possibly through E6*/p53 interaction (?), increase in cell adhesion by the activation of β-integrin signaling and overexpression of E-cadherin, decrease in tumor growth associated with a reduction in VEGFR-1; (**B**) Controversial effects of E6* in apoptosis regulation; and (**C**) Carcinogenic characteristics, such as: promotion of DNA damage by ROS, which may allow HPV DNA amplification and integration into the host genome, degradation of postsynaptic density-95/discs large/zonula occludens-1 domain (PDZ) containing proteins involved in cell polarity and adhesion and stabilization of E6 and E7 oncoproteins. The red T-bars indicate inhibition, the green arrows show induction of the related process, the black arrows depict an increment or a reduction in protein levels.

One of the most characterized E6* transcript functions is to facilitate translation of the E7 oncoprotein by increasing the space in the mRNA between the E6 stop codon and the E7 start codon, allowing better ribosome assembly [23,78,100]. However, other studies demonstrate that intron exclusion has a minimal or no effect on E7 translation, since the E7 protein is mainly translated from E6 non-spliced mRNA [22,101]. Moreover, other functions have been attributed to E6* proteins, mainly to E6*I, independent of E6 and E7 expression (Figure 4).

E6*I protein was detected for the first time in 1987 in CaSki cells [102] and like E6*II, displays both nuclear and cytoplasmic localization; conversely, E6 is mostly found in the cell nucleus [91,103,104].

E6*I HPV18 is a polypeptide of 57 aa that shares the first 44 aa of its N-terminal domain with E6 and contains 13 aa derived from the change in the E6 open reading frame after the splicing sites [25]. Due to different donor and acceptor splicing sites contained in HR-HPVs, the predicted E6*I proteins differ from E6, in size, by approximately 50 to 55 aa for HPV16, 18, 30, 33, 34, 36, 35, 39, 68 and 70; and 29 to 36 aa for HPV26, 31, 51, 56, 66, 69 and 82 [21].

The specific structure of E6*I has not been well characterized due to the difficulty in acquiring a compact monomeric fold in such a small polypeptide. However, α-helix or β-sheet conformations, depending on experimental conditions, have been suggested [105]. E6*I conserves only half of the N-terminal zinc binding motif present in E6. Moreover, most of the HR-HPV E6*I, excepting HPV56 and 66, contain a hydrophobic motif (L/M/I)XX(L/I/V)X(L/V/I) which is associated to E6 and E6AP binding [106].

It has been widely demonstrated that the HR-HPV E6 proteins promote p53 degradation through binding with the E3 ubiquitin ligase E6AP [107,108]. Furthermore, E6*I protein interferes with E6-mediated degradation of p53 by its binding to E6AP, E6 and to p53, although with lower affinity [21,91,109,110].

Furthermore, it has been shown that HPV18 E6 increases the levels of p14ARF through p53 degradation, while HPV18 E6*I over-expression only induces a moderate increase of the p14ARF [88]. This result shows that E6*I may have a direct effect over p14ARF, independent of E6, possibly through E6*I and p53 interaction, preventing p14ARF regulation by p53. However, more evidence is still needed.

Additionally, it has been demonstrated that HPV16 E6*I does not increase keratinocyte immortalization and proliferation [100]. HPV18 E6*I decreases cell proliferation in HPV16 positive cancer cells, while HPV18 E6*I overexpression in p53 null cancer cells does not exhibit this anti-proliferative effect, indicating that this effect could be attributed to protection of p53 by E6*I [110].

Anti-tumorigenic features have been associated with E6*I expression. The β-integrin pathway that regulates cytoskeleton rearrangements, cellular shape and mobility was evaluated in SiHa cells. The levels of β-integrin and its co-stimulatory molecule kindling-1, increased in the presence of E6*I, while a reduction in RhoA levels was observed, promoting cell morphological changes related to cell spreading. Moreover, this study found a decrease in Alkaline phosphatase activity in those cells transfected with HPV16 E6*I, which is related to loss of both pluripotency and undifferentiated cell phenotype [111].

Furthermore, in a study performed in SiHa cells, HPV16 E6*I promoted the overexpression of E-cadherin protein, a biological marker related to cell adhesion and epithelial phenotype. However, in C33A cells, this effect was not observed. Interestingly, a xenograft mouse model using SiHa and C33A cells transfected with HPV16 E6*I, showed an evident decrease in tumor size with a decrease in VEGFR-1 levels, a biological marker for angiogenesis [112].

Since E6*I does not induce immortalization and cell proliferation, it has been postulated that it could be regulating pathways involved in cell death, such as apoptosis. Different studies showed that both E6 and E6*I of HPV16 and HPV18 bind to the dead effector domain (DED) of procaspase 8 via different sites [113,114,115]; however, only HPV16 E6 can bind to Fas-associated protein with death domain (FADD) DED [116]. One of the studies showed that HPV16 E6*I stabilizes procaspase 8 while E6 has the opposite effect [114]; however, a further study demonstrates that neither HPV18 E6 nor E6*I induces procaspase 8 stabilization. Nevertheless, these viral proteins increase the levels of active caspase 8 and induce its nuclear translocation without inducing apoptosis [113].

Additionally, it has been shown that HPV16 E6 and E6*I exert different effects in apoptosis either together or alone. Both viral proteins independently expressed, promote resistance to TNF-induced apoptosis; in contrast, when they are expressed together they promote TNF-dependent apoptosis [109]. Furthermore, it has been demonstrated that overexpression of HPV16 E6*I but not E6*II, sensitizes oropharyngeal squamous cell carcinoma cell lines to radiation, promoting cell death [117]. Recent studies suggest that this effect could be dependent on cellular context, since it is not observed in non-head and neck cancer cell lines. Together, these facts indicate that the regulation of apoptosis by E6*I and E6 is a complicated mechanism and that the E6/E6* expression patterns and cellular contexts could play an important role.

A proteomic analysis comparing HPV16 positive and negative cell lines revealed that HPV16 E6*I modifies the expression of cellular proteins involved in a variety of cellular signaling pathways such as: integrin-linked kinase (ILK), oxidative phosphorylation and mitochondrial dysfunction. HPV16 E6*I promotes an increase in mitochondrial dysfunction in HPV positive and negative cells, which then induces a decrease in the levels of the antioxidant molecule GSH and subsequent DNA damage [111]. These data correlate with results observed in HPV16 positive cells, where HPV16 E6*I protein but not E6, decreases the levels of the antioxidant enzymes SOD2 and Gpx, leading to the accumulation of reactive oxygen species (ROS) and an increase in DNA damage [118]. Even when the DNA damage promoted by E6*I could eventually culminate in apoptosis, some data support the idea that the induction of DNA damage by ROS could be related to the amplification of HPV DNA, which would require different regulators of the homologous recombination DNA repair system [119] or to HPV genome integration [120], suggesting that E6*I could be participating in the HPV viral cycle, as well as in cancer establishment.

It is well known that E6 targets PDZ (postsynaptic density-95/discs large/zonula occludens-1 domain) containing proteins, inducing their degradation. Moreover, HPV18 E6*I protein, as well as E6, induces the degradation of PDZ containing proteins such as Dlg (Drosophila disc-large), MAGI-1 and h-Scrib. The ability to promote Dlg degradation is conserved among HPV31, 16 and 81 E6*I proteins; however, E6*I cannot bind to this protein. Currently, there is only one PDZ containing protein shown to interact with E6*I, allowing its degradation. This protein, termed PATJ can interact with E6*I in a PDZ binding motif (PBM)-independent manner or through other cell proteins that allow this interaction. In addition, this study demonstrated that HPV18 E6*I induces the degradation of Akt, in contrast to E6, which is not able to decrease Akt levels. This suggests that E6*I of HPV18 could be regulating processes involved in survival and cell growth [25,121].

Very little is known about the functions of other E6 spliced isoforms. HPV16 E6^E7 is a predominantly cytoplasmic protein that contains 41 aa of E6 in its N-terminal half and 38 aa of E7 in its C-terminal half. It has been shown that E6^E7 binds to the cellular chaperones HSP90α, HSP90β and Glucose-regulated protein 78 (GRP78) but only HSP90β and GRP78 induce E6^E7, E6 and E6*I stabilization. In addition, E6, E7 and E6*I proteins are stabilized by E6^E7, in a manner dependent on the endogenous chaperones [60].

## 8. Conclusions

The sustained higher proportion of E6*I compared to E6 mRNA observed in different lesions and tumors, suggests that the generation of E6* isoforms has an important role in cancer development.

Alternative splicing within the E6 ORF could be mediated by donor and acceptor splicing site sequences and surrounding fragments, which regulate the most efficient recruitment of the spliceosome elements. Discrepancies found in E6 splice patterns in diverse study models could be due to the presence of specific regulatory factors depending of the cell context or to differences in the physical state of the HPV genome during the progression of an HPV infection to cancer. The loss of E2 protein due to viral genome integration [122] could also affect the splicing process, since E2 is a mRNA binding protein which regulates E6 splicing [99]. Moreover, since HPV genome integration occurs at distinct sites in the host genome [75], it cannot be discarded that in some cases host genes involved in splicing regulation could be disrupted and therefore change the splicing patterns. Until now, little is known about the specific mechanisms regarding the modulation of E6 splicing patterns but all the evidence suggests that the presence of E6 spliced transcripts is a common event in cervical carcinogenesis.

It is worth mentioning that comparing the E6 splicing patterns among biological models analyzed with different methodological tools is a difficult task. The variations in results among different studies may be due to the choice of different techniques.

In studies using RT-PCR, the selection of primers commonly leads to the amplification of splice variants just within the E6/E7 ORFs, excluding some of the spliced transcripts involving the early HPV mRNA. In contrast, studies using deep sequencing techniques describe the splice forms extensively, allowing a robust analysis of the transcripts. Although this technology gives us a better approach to the diversity and quantity of E6 transcripts, more information is still needed to associate these transcripts with cancer progression. Moreover, it is difficult to achieve an adequate comparison between observations obtained through diverse methods that present different sensitivity. Nevertheless, the quality of the studies has increased over the time, permitting the detection of transcripts that are present in very low concentrations, such as E6^E7 (E6*X).

Some authors argue that while E6* transcripts can be abundant in some models, E6* proteins cannot be detected [59,101,123]. Nevertheless, other researchers have clearly identified E6* proteins, supporting that E6* transcripts can produce at least one E6* protein [91,109,114,118,124]. Anti-oncogenic effects have been attributed to E6* proteins [112], although other effects, such as promotion of DNA damage [118,120], degradation of PDZ containing proteins related to cellular polarity [25] and stabilization of E6 and E7 oncogenic proteins [60] are effects involved in cancer development, clearly demonstrated for E6* proteins . In addition, E6* proteins could have different effects depending on the cellular context where different E6* protein conformations could be generated [105], promoting distinct interactions with cellular binding partners.

The different splicing patterns for E6/E6* observed among tumors or during the different stages in cancer progression could provide a wide variety of E6 isoforms with an impact on biological processes. Nevertheless, oncogenic and/or non-oncogenic functions reported for E6* proteins, make it difficult to sort them out as tumor suppressor or oncoproteins in HPV-related tumors. Currently, the possibility that E6* proteins contribute to the HPV transformation process has gained attention and much data has been generated that has opened a window of opportunities in the study of these proteins regarding their participation in the HPV life cycle and/or in cancer establishment.

## Figures and Tables

**Table 1:** Transcripts derived from alternative splicing within the E6 open reading frame (ORF). The table summarizes the E6* isoforms for 23 HPV types where alternative splicing has been observed. The detailed donor and acceptor splicing sites for each E6 truncated transcript are enlisted below.

HPV Type	E6* Transcripts	Donor-Acceptor Splicing Sites (Nucleotide Position)	References
**16**	E6*I	226–409	[59]
	E6*II	226–526	[70]
	E6*III	226–3358	[59]
	E6*IV	226–2709	[71]
	E6*V	221–409	[61]
	E6*VI	191–409	[61]
	E6^E7 (E6*X)	226–742	[60]
	E6^E7*I	174–718	[62]
	E6^E7*II	221–850	[62]
**18**	E6*I	233–416	[63,64]
	E6*II	233–3434	[63,64]
	E6*III	233–2779	[64]
	E6^E7	233–791	[60,64]
**26**	E6*I	173–406	[21]
**30**	E6*I	229–420	[21]
**31**	E6*I	210–413	[65,68]
	E6^E4 (E6*III)	210–3295	[65]
**33**	E6*I	231–509	[66]
	E6*II	231–785	[66]
	E6*III	231–3351	[66]
**34**	E6*I	223–414	[21]
**35**	E6*I	228–419	[21]
**39**	E6*I	231–420	[21]
**45**	E6*I	230–413	[69]
**51**	E6*I	173–406	[21]
**52**	E6*I	224–502	[69]
**53**	E6*I	236–419	[69]
**56**	E6*I	157–420	[21]
**58**	E6*I	232–510	[67]
	E6*II	232–3355	[67]
**59**	E6*I	183–582	[69]
**66**	E6*I	157–420	[21]
**67**	E6*I	224–502	[69]
**68b**	E6*I	232–415	[69]
**69**	E6*I	178–411	[21]
**70**	E6*I	231–422	[21]
**73**	E6*I	227–410	[69]
**82**	E6*I	178–411	[21]
